# (Pentamethylcyclopentadienyl)chloridoiridium(III) Complex Bearing Bidentate Ph_2_PCH_2_CH_2_SPh-κ*P*,κ*S* Ligand

**DOI:** 10.3390/biom14040420

**Published:** 2024-03-30

**Authors:** Gerd Ludwig, Ivan Ranđelović, Dušan Dimić, Teodora Komazec, Danijela Maksimović-Ivanić, Sanja Mijatović, Tobias Rüffer, Goran N. Kaluđerović

**Affiliations:** 1 Institute of Chemistry, Martin Luther University Halle-Wittenberg, Kurt-Mothes-Straße 2, D-06120 Halle, Germany; ludwig.gerd7@gmail.com; 2 Department of Immunology, Institute for Biological Research “Sinisa Stankovic”—National Institute of the Republic of Serbia, University of Belgrade, Bulevar Despota Stefana 142, 11108 Belgrade, Serbia or randelovic.ivan@oncol.hu (I.R.); teodora.komazec@ibiss.bg.ac.rs (T.K.); nelamax@ibiss.bg.ac.rs (D.M.-I.); sanjamama@ibiss.bg.ac.rs (S.M.); 3 Department of Experimental Pharmacology, The National Tumor Biology Laboratory, National Institute of Oncology, Ráth György u. 7-9, 1122 Budapest, Hungary; 4 Faculty of Physical Chemistry, University of Belgrade, Studentski trg 12-16, 11000 Belgrade, Serbia; ddimic@ffh.bg.ac.rs; 5 Institute of Chemistry, Chemnitz University of Technology, Straße der Nationen 62, D-09111 Chemnitz, Germany; tobias.rueffer@chemie.tu-chemnitz.de; 6 Department of Engineering and Natural Sciences, University of Applied Sciences Merseburg, Eberhard-Leibnitz-Strasse 2, D-06217 Merseburg, Germany

**Keywords:** iridium(III), pentamethylcyclopentadienyl ligand, in vitro, anaplastic thyroid tumor 8505C, apoptosis

## Abstract

The (pentamethylcyclopentadienyl)chloridoiridium(III) complex bearing a κ*P*,κ*S*-bonded Ph_2_PCH_2_CH_2_SPh ligand ([Ir(η^5^-C_5_Me_5_)Cl(Ph_2_P(CH_2_)_2_SPh-κ*P,*κ*S*)]PF_6_, (**1**)] was synthesized and characterized. Multinuclear (^1^H, ^13^C and ^31^P) NMR spectroscopy was employed for the determination of the structure. Moreover, SC-XRD confirmed the proposed structure belongs to the “piano stool” type. The Hirshfeld surface analysis outlined the most important intermolecular interactions in the structure. The crystallographic structure was optimized at the B3LYP-D3BJ/6-311++G(d,p)(H,C,P,S,Cl)/LanL2DZ(Ir) level of theory. The applicability of this level was verified through a comparison of experimental and theoretical bond lengths and angles, and ^1^H and ^13^C NMR chemical shifts. The Natural Bond Orbital theory was used to identify and quantify the intramolecular stabilization interactions, especially those between donor atoms and Ir(III) ions. Complex **1** was tested on antitumor activity against five human tumor cell lines: MCF-7 breast adenocarcinoma, SW480 colon adenocarcinoma, 518A2 melanoma, 8505C human thyroid carcinoma and A253 submandibular carcinoma. Complex **1** showed superior antitumor activity against cisplatin-resistant MCF-7, SW480 and 8505C cell lines. The mechanism of tumoricidal action on 8505C cells indicates the involvement of caspase-induced apoptosis, accompanied by a considerable reduction in ROS/RNS and proliferation potential of treated cells.

## 1. Introduction

Cisplatin has been widely used as the “gold standard” in cancer treatment for over 60 years [1,2,3,4]. Despite its success in treating various types of cancers, cisplatin has certain limitations, including the development of resistance and severe toxic effects, such as kidney, ear, and brain damage [5,6,7]. Platinum-based drugs, such as cisplatin, carboplatin, and oxaliplatin, have been widely used as effective chemotherapeutic agents in cancer treatment. Nevertheless, they exhibit limitations due to side effects which have led to research focusing on the development of transition metal-based drugs as alternatives to cisplatin and other platinum-based anticancer drugs [8,9]. Utilizing different metals in cancer therapy can potentially affect tumor cells differently and induce distinct molecular pathways compared to platinum-based drugs. Non-platinum-based compounds, including titanium, ruthenium, tin, palladium, and gold complexes, are in focus as potential anticancer agents [9,10,11,12,13,14,15]. 

Iridium complexes offer unique chemical and physical properties that can be advantageous for developing new antitumor agents [9,16], and because of that, iridium-based drugs have gained attention as potential alternatives. One notable advantage of iridium-based drugs is their ability to exhibit different coordination geometries and binding modes compared to platinum. This structural diversity allows for the fine-tuning of their interactions with cellular targets, potentially leading to improved selectivity and reduced side effects [17]. Additionally, iridium complexes have shown the ability to overcome resistance mechanisms observed with platinum-based drugs, providing an alternative treatment option for resistant tumors [17,18,19]. 

Most studies showed an improved uptake of iridium(III) complexes by tumor cells and a high affinity to lysosomes, mitochondria and the endoplasmic reticulum. The platform of the antitumor effect of iridium(III) complexes differs from cisplatin. The main disruption in cell function promoted by iridium-based compounds is mediated by mitochondrial damage, endoplasmic reticulum stress and impaired redox status, and down-regulates energy metabolism, leading to a reduction in cell proliferation, invasion and metastasis. In addition, some iridium(III) complexes can also cause immunogenic cell death, which promotes host defense against tumors by refreshing immune cell function [17,18,19]. All these interactions may be the reason why iridium-based complexes can overcome chemotherapy resistance, as they are not affected by the upregulated DNA repair, multidrug-resistant pumps, expression of anti-apoptotic molecules, etc., which are established as cell resistance to conventional chemotherapeutics [20]. 

In particular, (cyclopentadienyl)iridium(III) complexes and their derivatives have attracted researchers as promising anticancer agents [21,22,23,24]. These complexes exhibit strong electron-donating properties, promote rapid ligand exchange, and form aqua complexes easily [23]. They might also generate low levels of reactive oxygen species and impact mitochondrial membrane potential. Additionally, half-sandwich complexes provide chemical structure varieties with the modulation over the hydrophobic characteristics of the cyclopentadienyl moiety. Half-sandwich (cyclopentadienyl)iridium(III) compounds are found to exhibit potent antitumor effects. Complexes of the type [Ir(η^5^-C_5_Me_5_)Cl(X^∩^Y)]*^n^*^+^ (X^∩^Y = bipyridine/phenanthroline/2-phenylpyridine) exhibited very high activity against various tumor cells in the lower µM concentrations [24,25,26]. Half-sandwich naphthalimide-modified iridium(III) complexes demonstrated very good photophysical properties as well as expressed high anticancer activity in HeLa, A549 and HepG2 cancer cells [27]. The mode of cell death induced with these organoiridium(III) complexes is related to the induction of apoptosis. [Ir(η^5^-C_5_Me_5_)Cl(P^∩^P)]PF_6_ (P^∩^P = 2,20-*bis*(diphenylphosphino)-1,10-bi-naphthyl) showed better antitumor activity than cisplatin against HeLa and A549 cancer cells [28]. This complex interacts with ctDNA, however no binding to 9-Me-adenine and 9-Et-guanine could be confirmed. Thus, it was assumed that interaction with DNA may not be the main mode of action. [Ir(η^5^-C_5_Me_5_)Cl(P^∩^P)]PF_6_ induces apoptosis and upregulates ROS in A549 cells as well as interacts with coenzyme NAD^+^/NADH couple. However, some of the prepared iridium(III) complexes bearing pentamethylcyclopentadienyl moiety and bidentate ligands such as ethylendiamine, 2,2′-bipyridine, 1,10-phenanthroline or monodentate 1,3,5-triaza-7-phosphaadamantane) had no impact on tumor cells in in vitro conditions [29,30].

**Figure 1:**
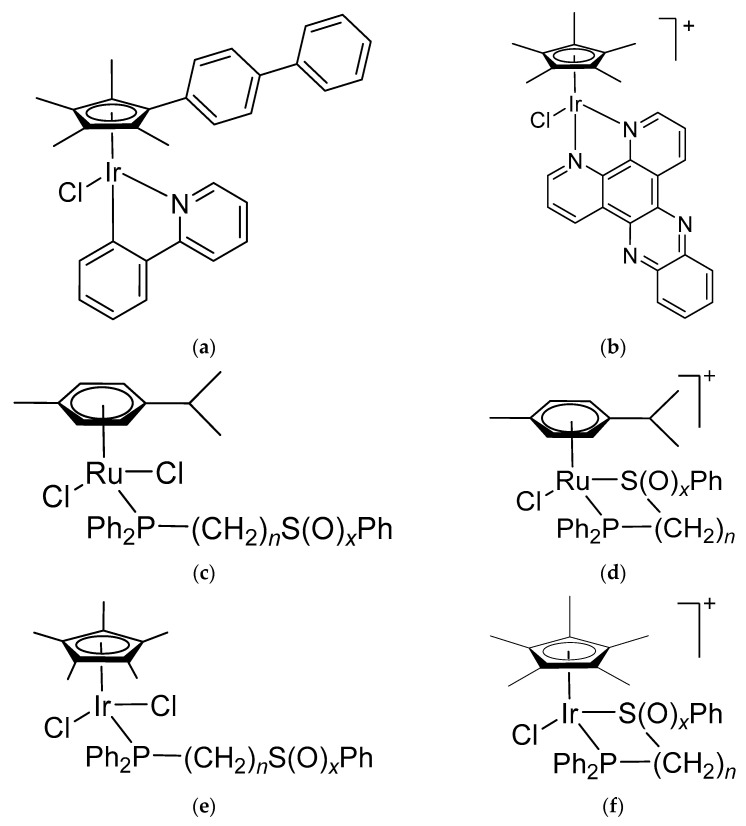
Various iridium(III) (**a**,**b**) and ruthenium(II) complexes (structures: (**c**,**d**) *x* = 0–2; *n* = 1–3; (**e**,**f**) *x* = 0–2; *n* = 1, 3) with high anticancer activity.

Recently we reported on the (η^6^-arene)ruthenium(II) (arene = benzene, mesitylene, indane, 1,2,3,4-tetrahydronaphthalene, and 1,4-dihydronaphthalene) and (η^5^-C_5_Me_5_)Ir(III) complexes containing Ph_2_P(CH_2_)*_n_*S(O)*_x_*Ph (*n* = 1–3; *x* = 0–2) with κ*P*- and κ*P*,κ*S*-coordination (Figure 1) [31,32,33,34,35,36]. Those complexes expressed high anticancer potential in the lower µM range. Moreover, they were found more efficiently compared to cisplatin against A253, 518A2, SW480, 8505C and MCF-7 tumor cell lines. For several complexes, mechanism of action studies demonstrated the induction of apoptotic mode of cell death. From this series of iridium(III) complexes we described those with κ*P*- and κ*P*,κ*S*-Ph_2_P(CH_2_)*_n_*S(O*_x_*)Ph ligands (*x* = 0–2) with methylene (*n* = 1) and propylene spacers (*n* = 3) between P and S atoms. Herein, we report on the preparation, characterization, and theoretical analysis of (pentamethylcyclopentadienyl)iridium(III) complex with Ph_2_PCH_2_CH_2_SPh-κ*P*,κ*S* ligand. In vitro activities against human cell lines from different origins, namely MCF-7, SW480, 518A2, 8505C and A253, were investigated. Furthermore, the mechanism of action against 8505C cell lines was determined.

## 2. Materials and Methods

### 2.1. General Comments

All reactions and handling procedures were conducted in an argon atmosphere using established Schlenk techniques. Methanol was subjected to a drying procedure (over magnesium) and freshly distilled before application. NMR spectra (^1^H, ^13^C and ^31^P) were acquired at 27 °C using Varian Gemini 200 and VXR 400 spectrometers (Palo Alto, USA). Chemical shifts (δ) are reported in parts per million (ppm) with respect to residual solvent signals (CD_2_Cl_2_: δ_H_ = 5.32 ppm, δ_C_ = 53.8 ppm); δP values are referenced to H_3_PO_4_ (85%) as an external standard. Elemental analysis (C, H) was conducted at the Microanalytical Laboratory of Martin Luther University Halle-Wittenberg in Germany using a CHNS-932 (LECO) elemental analyzer (St. Joseph, USA). Ph_2_P(CH_2_)_2_SPh and [{IrCl_2_(η^5^-C_5_Me_5_)}_2_] were synthesized following literature protocols [37,38].

### 2.2. Preparation of [IrCl_2_(η^5^-C_5_Me_5_){Ph_2_P(CH_2_)_2_SPh-κP,κS}]PF_6_ (***1***)

To a methanol solution (30 mL) of [{IrCl_2_(η^5^-C_5_Me_5_)}_2_] (127 mg, 0.16 mmol) Ph_2_P(CH_2_)_2_SPh (0.32 mmol) was added while stirring. The solution was heated under reflux for 3 h, afterward [NH_4_][PF_6_] (6 equiv.) was added. Upon cooling (–70 °C) obtained precipitate was filtered off, washed with diethyl ether (3 × 2 mL), and dried in a vacuum. Yield: 172 mg (65%). Anal. Found: C, 43.57; H, 4.15. Calcd for C_30_H_34_ClF_6_IrP_2_S (830.27): C, 43.40; H, 4.13. 

^1^H NMR (400 MHz, CD_2_Cl_2_): δ 1.50 (d, ^4^*J*_P,H_ = 2.40 Hz, 15H, CH_3_), 2.87 (m, 1H, C*H*_2_PPh_2_), 3.13 (m, 1H, C*H*_2_PPh_2_), 3.66 (m, 1H, SC*H*_2_), 4.20 (m, 1H, SC*H*_2_), 7.12–7.70 ppm (m, 15H, HPh).

^13^C NMR (100 MHz, CD_2_Cl_2_): δ 8.1 (d, ^3^*J*_P,C_ = 0.7 Hz, CCH_3_), 25.2 (d, ^1^*J*_P,C_ = 31.6 Hz, CH_2_PPh_2_), 34.7 (s, SCH_2_), 96.9 (d, ^2^*J*_P,C_ = 2.4 Hz, CCH_3_), 123.7–135.5 ppm (CPh). ^31^P NMR (162 MHz, CD_2_Cl_2_): δ 30.6 (s), –144.5 (sept, ^1^*J*_P,F_ = 709.8 Hz, PF_6_).

### 2.3. X-ray Crystallography

Data of **1** were collected using a Rigaku Oxford Gemini S diffractometer at 120 K. The structures were determined through direct methods and subjected to refinement using full-matrix least-squares procedures on *F*^2^ with SHELXS/SHELXL-2013 [39]. Anisotropic refinement was applied to all non-hydrogen atoms, while C-bonded hydrogen atoms were refined using a riding model. Detailed crystal and structural refinement data of **1** can be found in Table S1. The complete dataset is available in the Cambridge Crystallographic Data Centre (CCDC) under accession code 2330744, and interested parties can obtain copies of the data free of charge through https://www.ccdc.cam.ac.uk/structures/, accessed on 25 March 2024.

### 2.4. Hirshfeld Surface Analysis

The theoretical analysis of the crystallographic structure was performed in the CrystalExplorer program package [40]. This type of analysis is important for the overall stability of the crystal package and it can be used for the determination of contributions of specific interactions to the stability. Hirshfeld surface analysis is represented by a graph connecting two distances, the first one being the distance between the two nearest nuclei (de) and the second distance representing the distance between nuclei and the external surface (di) [41,42,43]. These distances are colored in red, white, and blue if the values are shorter, equal, or longer than the van der Waals radii. The normalized distances in this contribution are shown between –X (red) and Y (blue). Fingerprint plots show the spatial distribution and percentages of specific interatomic interactions. The most important atom connections are shown in the Supplementary Materials. 

### 2.5. Theoretical Analysis of Structure

The structure of the complex was optimized in the Gaussian 09 Program Package [44] starting from the crystallographic structure at B3LYP-D3BJ/6-311++G(d,p)(H,C,P,S,Cl)/LanL2DZ(Ir) level of theory [45,46,47,48,49]. The optimization was performed without any geometrical constraints and the obtained geometry was proven to be a minimum on the energy surface by the absence of imaginary frequencies. The Conductor-like Polarizable Continuum (CPCM) model was applied for the optimization of the compound in the solvent used for NMR spectroscopy [50]. The chemical shieldings were obtained by the Gauge Independent Atomic Orbital Approach (GIAO) [51]. The chemical shifts were calculated relative to TMS optimized at the same level of theory. The intramolecular interactions governing stability were investigated by the Natural Bond Orbital Approach [52] as implemented in the Gaussian Program Package. 

### 2.6. In Vitro Investigations

#### 2.6.1. General Remarks

Dimethyl sulfoxide (DMSO), phosphate-buffered saline (PBS), RPMI-1640 medium, fetal calf serum (FCS), and propidium iodide (PI) were sourced from Sigma (St. Louis, MO, USA). Annexin V-FITC (AnnV) was procured from Biotium (Hayward, CA, USA), while Acridine orange (AO) was obtained from Labo-Moderna (Paris, France). Apostat, CFSE (carboxyfluorescein succinimidyl ester) and DHR (dihydrorhodamine 123) were acquired from R&D Systems (Minneapolis, MN, USA). Human thyroid carcinoma 8505C, submandibular gland carcinoma A253, breast adenocarcinoma MCF-7, melanoma 518A2, colon cancer SW480 and primary fetal lung fibroblast MRC-5 cell lines were obtained from ATCC. The cells were routinely cultured in nutrient medium: RPMI-1640 medium, HEPES-buffered and supplemented with 10% FCS, 2 mM L-glutamine, 0.01% sodium pyruvate, 5 × 10^−5^ M 2-mercaptoethanol, and antibiotics (referred to as culture medium) at 37 °C in a humidified atmosphere with 5% CO_2_. Following standard trypsinization, cells were plated at a density of 1 × 10^4^ to 2.5 × 10^3^ cells per well in 96-well plates for viability assessments (for MRC-5 1 × 10^4^ cells per well were used) and 1.5 × 10^5^ cells per well in 6-well plates for flow cytometry analyses. Stock solutions of the tested compounds were prepared in DMSO at a concentration of 20 mM and then further diluted with a nutrient medium to achieve various working concentrations. 

#### 2.6.2. SRB Assay

For viability assay standard procedure was used as described [53]. The final concentrations of **1** or cisplatin applied ranged from 0.78 to 50.0 µM on tumor cells, while 3.12 to 200.0 µM concentrations were used in the case of MRC-5 cells. Treatments of the investigated cells were performed for 96 h. Experiments were achieved in triplicates.

#### 2.6.3. Flow Cytometry Analyses

8505 cells were treated (72 h) with IC_50_ concentration of **1** and PI for cell cycle perturbation, annexin V/PI, apostat, AO, CFSE and DHR assays were performed as previously described [33,54].

## 3. Results and Discussion

### 3.1. Preparation and Characterization of [Ir(η^5^-C_5_Me_5_)Cl(Ph_2_P(CH_2_)_2_SPh-κP,κS)][PF_6_] (***1***)

**Scheme 1:**
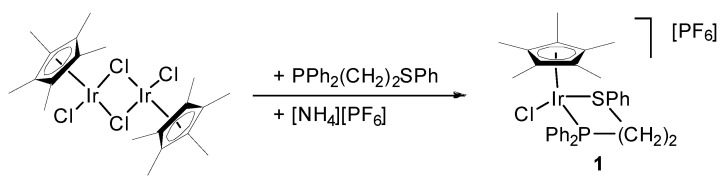
Preparation of [Ir(η^5^-C_5_Me_5_)Cl{Ph_2_P(CH_2_)_2_SPh-κ*P*,κ*S*}][PF_6_] (**1**).

The cationic iridium(III) complex (**1**) was prepared according to Scheme 1. Thereby, dimeric [Ir(η^5^-C_5_Me_5_)Cl_2_}_2_] was cleaved along the Ir–Cl–Ir bridges by the formation of yellow and mononuclear **1**. Thus obtained **1** was characterized by elemental analysis, multinuclear (^1^H, ^13^C, and ^31^P) NMR spectroscopy, and single-crystal X-ray structure analysis. Elemental analysis verified the composition of **1** and confirmed its purity.

### 3.2. Molecular Structure of ***1***

**Figure 2:**
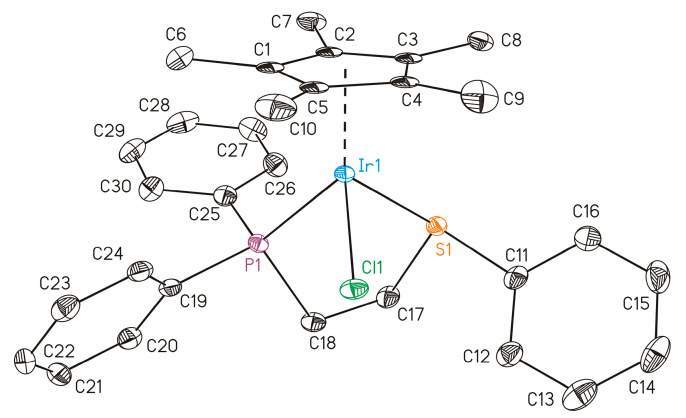
Molecular structure of [Ir(η^5^C_5_Me_5_)Cl{κ*P*,κ*S*-Ph_2_P(CH_2_)_2_SPh}]^+^ (**1a**, ellipsoid probability 50%, H atoms omitted for clarity). Structural parameters (distances in Å, angles in °): Ir1–Cl1 2.393(1), Ir1–P1 2.293(1), Ir1–S1 2.350(1), P1–Ir1–S1 84.45(4), Cl1–Ir–P1 87.42(4), Cl1–Ir–S1 91.77(4), C11–S1–C17 102.9(2), C19–P1–C25 104.7(2), C19–P1–C18 103.6(2), C25–P1–C18 106.6(2).

Organoiridium(III) complex **1** crystallized (triclinic crystal system, space group P$\bar{1}$) from the acetone/n-pentane solution at room temperature. The unit cell of **1** consists of an iridium(III) complex cation, [Ir(η^5^C_5_Me_5_)Cl{Ph_2_P(CH_2_)_2_SPh-κ*P*,κ*S*}]^+^ (**1a**), a PF_6_^–^ anion, and one acetone packing solvent molecule. The structure of the complex is depicted in Figure 2, while specific structural parameters can be found in the respective figure caption and Tables S1 and S2. The main structural feature of **1a** is a half-sandwich—“piano stool” structure. The iridium(III) ion is surrounded by an η^5^-C_5_Me_5_ ligand (with an Ir∙∙∙ring centroid distance of 1.846 Å), a chlorido and a bidentate Ph_2_P(CH_2_)_2_SPh-κ*P*,κ*S* ligands. In the cation of **1**, [Ir(η^5^C_5_Me_5_)Cl{Ph_2_P(CH_2_)_2_SPh-κ*P*,κ*S*}]^+^ (**1a**), the angles around the iridium(III) atom due to the κ*P*,κ*S* coordination of the ligand are ranging from 84.45(4)° to 91.77(4)° (Table S2), making the structure a distorted octahedron. The five-membered iridacycle [IrPC_2_S] in **1a** adopts a half-chair conformation. The bond lengths of Ir–Cl (2.392(1) Å), Ir–P (2.293(1) Å) and Ir–S (2.350(1) Å) are within the expected ranges (median Ir–Cl: 2.419 Å, lower/higher quartile: 2.315/2.606 Å, *n* = 543; median Ir–P: 2.316 Å, lower/higher quartile: 2.151/2.462 Å, *n* = 543; median Ir–S: 2.357 Å, lower/higher quartile: 2.244/2.423 Å, *n* = 148; *n* denotes the number of observations [34]). Similar bond lengths were found in previous contributions for Ir–Cl (2.402(9) Å), Ir–P (2.310(1) Å) and Ir–S (2.357(1) Å) [34]. A certain relaxation of the mentioned structure is possible due to the presence of (-CH_2_-)_3_ chain.

### 3.3. Hishfeld Surface Analysis

The Hirshfeld surface analysis is important for the investigation of the stabilization interactions within crystal structures. These interactions are governed by the present structural parameters and could imply future synthesis and change in substituents that influence the interactions biomolecules. As described in Section 3.2, **1a** contains pentamethylcyclopentadienyl and chlorido ligands as well as κ*P*,κ*S*-Ph_2_P(CH_2_)_2_SPh-coordinated molecule that surround iridium(III) ion. The abundance of carbon and hydrogen atoms and their position surrounding other elements limits the possible interactions between units in a crystal package. The unit also contains PF_6_^−^ and acetone which can also be important for the overall stabilization of structure through interactions with the complexes from surrounding cells. The fingerprint plots for the specific interactions are given in the Supplementary material.

Due to the abundance of hydrogen atoms in structures of pentamethylcyclopentadienyl and κ*P*,κ*S*-Ph_2_P(CH_2_)_2_SPh ligand, the main contribution to stabilization interaction originates from H∙∙∙H contacts (49.7%). The importance of the counter ion for the formation of structure can be seen in the relative amount of H∙∙∙F contacts (27.1%). These interactions are formed between counter ion and iridacycle [IrPC_2_S]. The interactions between hydrogen and chlorido ligands contribute to the overall contacts for 3.6% which is higher compared to other interactions. The interactions denoted as H∙∙∙O (2.7%) include stabilization interactions formed between hydrogen atoms of cyclic moieties and acetone, as this is the only compound containing oxygen. This also verifies the importance of co-crystalized solvent molecules for stability. The C∙∙∙H contacts account for 13.8% of interactions that include H∙∙∙π contacts. This is also expected bearing in mind the presence of π-delocalized electrons in the structure of the complex. Other interactions including carbon atoms contribute by lower percentages, for example, C∙∙∙C (1.8%), C∙∙∙F (0.3%), and C∙∙∙O (0.3%). It is important to outline that other heteroatoms, such as phosphorus and sulfur do not contribute to the stabilization interactions as they are sterically hindered and distant from the other unit cells. The central metal ion is completely surrounded by the ligands and no interactions involving this ion were observed, as expected. The specific intermolecular interactions governing stability of the compound are examined in the following section. Based on Hirshfeld surface analysis, it can be concluded that heteroatoms (P and S) and central metal ions are not crucial for the interactions with surrounding units, for example, proteins and DNA, but they influence the overall geometry of the compound. Other groups, such as ring structures are most certainly included in interactions, through various π–π and π–alkyl bonds.

### 3.4. Theoretical Analysis

**Figure 3:**
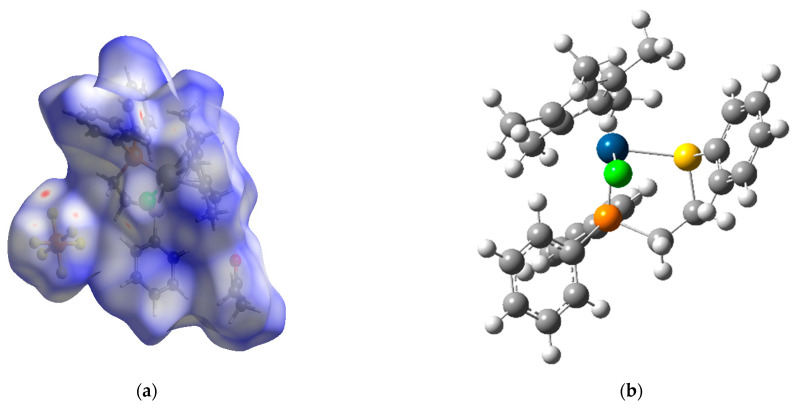
(**a**) Hirshfeld surface map of the obtained crystal and (**b**) optimized complex structure (at B3LYP-D3BJ/6-311++G(d,p)((H,C,P,S,Cl)/LanL2DZ(Ir) level of theory) (Hydrogen-white, Carbon-gray, sulfur-yellow, phosphorus-orange, iridium-blue.

Upon optimization, the structure was compared to the crystallographic one by calculating the mean absolute error (MAE) and correlation coefficient (R) between the theoretical and experimental bond lengths and angles given in Tables S1 and S2. The parameters were selected in order to verify the applicability of the B3LYP-D3BJ/6-311++G(d,p)(H,C,P,S,Cl)/LanL2DZ(Ir) level of theory. The same level of theory was previously used to examine the structural and catalytic properties of Ir compounds [55,56]. The optimized structure is shown in Figure 3.

When experimental and theoretical bond lengths and angles were compared, high correlation coefficients (>0.99) were obtained. The MAE values for bond lengths and angles were 0.01 Å and 1.0°, respectively. These values are of the order of experimental error, which proves the assumption that the optimized structure represents well the experimental one. The distance between carbon atoms of pentamethylcyclopentadienyl moiety and Ir is between 2.193(4) and 2.234(4) Å in crystallographic and between 2.21 and 2.28 Å in optimized structure (Table S2). On the other hand, the distance between Ir and Cl atoms is 2.393(1) (experimental) and 2.45 Å (theoretical). Two other positions in the vicinity of central metal ion are occupied by P (2.293(1) experimental and 2.31 Å optimized) and S atoms (2.350(1) experimental and 2.42 Å theoretical). For each of the mentioned bond lengths slightly higher values were observed in theoretical vs. experimental values, which is a consequence of the system relaxation upon optimization. Also, it should be kept in mind that the optimization was performed for the isolated compound in vacuum, while in crystallographic structure additional interactions were responsible for the stabilization of the system, as explained in the previous section. For all other bond lengths much lower differences were observed between experimental and theoretical. For instance, in κ*P*,κ*S*-Ph_2_P(CH_2_)_2_SPh as well as pentamethylcyclopentadienyl ligands are characterized by the extended delocalization of electron density. Due to the different donating abilities of the ligands (e.g., pentamethylcyclopentadienyl: π-donor; chlorido: σ-donor), the bond angles deviate from the ideal octahedral geometry, although a high resemblance was obtained between experimental and theoretical values (Table S3). The bond angle between S1–Ir1–Cl1 is 91.77(4)° in crystal structure and 91.9° in optimized structure. Similar was observed for Cl1–Ir1–P1 (87.42(4)° in solid state and 86.9° in optimized structure). An additional reason for deviation from expected values is due to the fact that sulfur and phosphorus atoms are part of the same ligand system. The angle S1–C17–C18 of the iridacycle [IrPC_2_S] is 113.0(3)° in experimental and 113.6° in theoretical structure, while the same angles for C20–C19–P1 part are 119.5(4) and 120.2°, respectively. The rest of the angles are within the expected range. 

Due to the presence of various donor atoms, it is beneficial to analyze the intramolecular interactions of the investigated complex. Some of the representative stabilization interactions are listed in Table S4. The most numerous stabilization interactions are formed within phenyl and pentamethylcyclopentadienyl moieties, as expected from a structural point of view [57,58]. These interactions, denoted as π(C–C)_arom_→π*(C–C)_arom_, are much stronger in the case of aromatic rings with delocalization of π-electrons and stabilization energies between 82.3 and 98.0 kJ mol^−1^. Pentamethylcyclopentadienyl moiety is characterized by interactions with a stabilization energy of 8.9 kJ mol^−1^. The complex structure is stabilized by the electron donation from pentamethylcyclopentadienyl to Ir, as this is the most stable part of the structure. Several interactions are worth mentioning. The stabilization interaction can be formed by electron donation from C–C bonds to the empty orbitals of Ir(III), denoted as π(C–C)→LP*(Ir), with stabilization interactions between 19.9 and 88.9 kJ mol^−1^, depending on the type of C–C bond that is included in interaction. Additionally, interactions occur between C–C and C–Ir bonds, π(C–C)→ σ*(Ir–C), with stabilization interactions between 67.5 and 370 kJ mol^−1^. These values prove the importance of the aromatic structure for the stabilization of central metal ions. The electron donation from C–C bonds of pentamethylcyclopentadienyl moiety is important for the stabilization of interactions formed between other donor atoms and Ir, such as π(C–C)_arom_→σ*(Ir–P) (14.5 kJ mol^−1^) and π(C–C)_arom_→σ*(Ir–S) (6.2 kJ mol^−1^). The interaction formed between carbon atoms and Ir also stabilizes other interactions between donor atoms and Ir, for example σ(Ir–C)→σ*(Ir–C) (125.1 kJ mol^−1^), σ(Ir–C)→σ*(Ir–P) (44.8 kJ mol^−1^), and σ(Ir–C)→σ*(Ir–S) (123.8 kJ mol^−1^). The lone pair on sulfur is included in several interactions, such as with the lone pair of central metal ion (LP(S)→LP*(Ir), 3.9 kJ mol^−1^) or with Ir–C bond (LP(S)→σ*(Ir–C), 4.9 kJ mol^−1^). The lone pair of sulfur additionally stabilizes the structure of the ligand through a donation to neighboring C-C bonds of the aromatic ring (LP(S)→π*(C–C), 22.7 kJ mol^−1^). As a lone pair of phosphorus is already included in the interaction formation with central metal ions, there are no additional interactions with the surrounding groups. The presence of chlorido ligand and its lone pairs stabilize the surrounding interactions, as shown in Table S4. Lone pairs of iridium interact with the surrounding bonds through LP(Ir)→π*(C–C), LP(Ir)→σ*(C–S), and LP(Ir)→σ*(C–P) interactions characterized by the stabilization energies of 48.1, 6.0, and 5.8 kJ mol^−1^, respectively. These results can be beneficial when other organoiridium complexes are synthetized, as they justify the importance of selected ligand systems. 

### 3.5. Experimental and Theoretical NMR Characterization

The prediction of NMR spectra was performed for further structural analysis and compared to experimental data. The theoretical chemical shifts were calculated from the chemical shieldings relative to TMS optimized at the same level of theory. These values were overestimated and a correction factor (0.995 for ^1^H and 0.912 for ^13^C chemical shifts) was determined after the comparison of experimental and theoretical values. Table 1 presents these two sets of values. 

In the ^1^H NMR spectra of complex **1** the lowest chemical shifts were obtained for the methyl hydrogen atoms (1.50 and 1.69 ppm in experimental and theoretical spectra, respectively). Due to the unequal chemical surrounding two protons attached to the carbon atom adjacent to PPh_2_ have different chemical shifts (2.87 and 3.13 in the experimental spectrum). These values differ for 0.31 and 0.01 ppm from the calculated values. In the presence of another electronegative element, chemical shifts of hydrogen atoms of the SCH_2_ group have higher chemical shifts, as seen in Table 1. Hydrogen atoms from the phenyl groups resonate in the range between 7.12 and 7.70 ppm (experimental value (theoretical: 7.06 and 8.18 ppm). The experimental values are well reproduced with a correlation coefficient of 0.97 and MAE of 0.3 ppm. In the ^13^C NMR spectrum, the lowest chemical shifts were observed for methyl carbon atoms of pentamethylcyclopentadienyl moiety. Again, the carbon atoms bonded to phosphorus and sulfur are more shielded in the experimental (25.2 and 34.7 ppm) than in the predicted (36.2 and 40.8 ppm) spectrum. The discrepancy between experimental and theoretical chemical shift values of *C*H_2_PPh_2_ is a consequence of the solvent effect and high electronegativity of phosphorus. The chemical shifts of pentamethylcyclopentadienyl carbon atoms are almost the same in both spectra (96.9 vs. 96.3 ppm). Carbon atoms of the phenyl group showed an expected pattern between 123.7 and 135.5 ppm in the experimental spectrum, which coincides well with the range of theoretical values (121.9–132.6 ppm). The theoretical values resemble the experimental ones to a high degree with a correlation coefficient of 0.99 and an MAE value of 4.1 ppm.

### 3.6. Effect of ***1*** on the Viability of Tumor Cells

**Figure 4:**
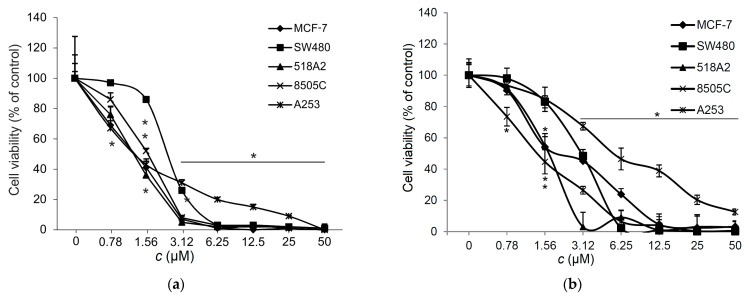
Tumor cell viability in response to the **1** (**a**) and cisplatin (**b**) measured by SRB assay (treatment, 96 h). Statistical significance * *p* < 0.05.

To determine the biological potential of **1**, in vitro cytotoxicity studies were performed against MCF-7 breast adenocarcinoma, SW480 colon adenocarcinoma, 518A2 melanoma, 8505C human thyroid carcinoma and A253 submandibular carcinoma cell lines, as well as toxicity was assessed on primary human fibroblasts MRC-5. The cells were cultured in the presence of various concentrations of **1** or corresponding conventional chemotherapeutic—cisplatin and the cell viability was analyzed after 96 h by the sulforhodamine-B (SRB) microculture colorimetric assay [59]. According to the obtained results, both, **1** as well as cisplatin induced a dose-dependent decrease in cell viability (Figure 4). The IC_50_ values, along with those of cisplatin and relevant iridium(III) and ruthenium(II) complexes bearing ω-diphenylphosphino-alkyl phenyl sulfide ligands, Ph_2_P(CH_2_)*_n_*SPh-κ*P*,κ*S* (*n* = 1, 3), as well as Ph_2_P(CH_2_)_2_SPh, for comparison, are shown in Table 2. Upon coordination of phosphine-sulfide ligand to iridium(III) cation an enhancement of activity (4.5–15.8 times) is observed. In general, the activity of **1**, having ethylene spacer between P and S atoms, against all cell lines lays between iridium(III) complexes of the same type with methylene and propylene spacers, thus [Ir(η^5^C_5_Me_5_)Cl{Ph_2_P(CH_2_)*_n_*SPh-κ*P*,κ*S*}][PF_6_] (*n* = 1 or 3) (Table 2). Analogous ruthenium(II) complex bearing the same phospino-sulfide ligand also in a bidentate fashion, [Ru(p-cym)Cl{Ph_2_P(CH_2_)_2_SPh-κ*P*,κ*S*}][PF_6_], showed a similar effect on 518A2 and 8505C as **1**; however, its activity was higher on MCF-7, SW480 and A253 cell lines. In direct comparison with cisplatin, complex **1** showed superior activity against MCF-7, SW480 and 8505C cell lines, but similar or lower against 518A2 and A253 cells, respectively. Importantly, evaluation of complex **1** selectivity (Table 2, Figure S2) toward malignant phenotype revealed a significant advantage of a newly designed drug in comparison to conventional therapeutics, with a selectivity index varying from 9 to 12.

### 3.7. Complex ***1*** Triggers Apoptosis in 8505C Cells

**Figure 5:**
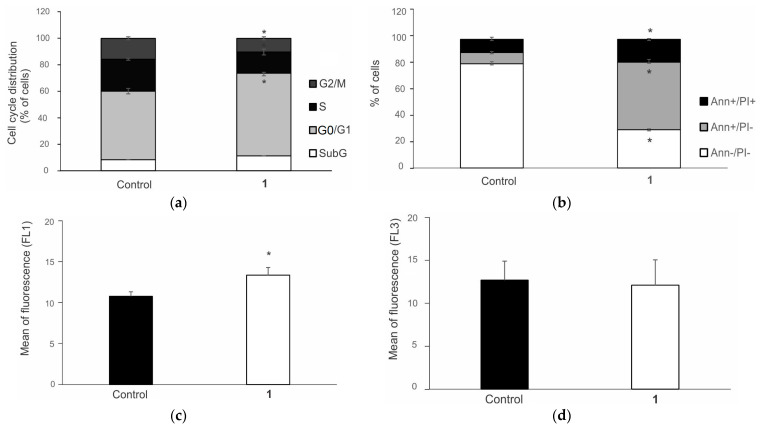
Effect of **1** on the (**a**) cell cycle distribution, (**b**) induction of apoptosis, (**c**) caspase activation and (**d**) autophagic process in 8505C cells. Statistical significance * *p* < 0.05.

Due to similar antiproliferative activity of **1** on MCF-7, SW480 and 8505C, and in order to compare with similar iridium(III) and ruthenium(II) complexes, the mechanism of action was studied on thyroid carcinoma 8505 cell line. At first, the cell cycle distribution of the 8505C cell line under treatment (72 h) with **1** (IC_50_ concentration) was investigated (Figure 5a). Complex **1** enhanced the accumulation of cells in the sub-G1 and G0/G1 phases, while decreasing the DNA synthesis rate. The impact of **1** on the distribution of the cell cycle appears to resemble that of the previously evaluated iridium(III), [Ir(η^5^C_5_Me_5_)Cl_2_{Ph_2_PCH_2_S(O)Ph-κ*P*}] and [Ir(η^5^C_5_Me_5_)Cl{Ph_2_P(CH_2_)_3_S(O)Ph-κ*P*,κ*S*}][PF_6_], and ruthenium(II) complexes, [Ru(p-cym)Cl_2_{Ph_2_P(CH_2_)_3_S(O)*_x_*Ph-κ*P*}] and [Ru(p-cym)Cl{κ*P*,κ*S*-Ph_2_P(CH_2_)_3_S(O)*_x_*Ph-κ*P*,κ*S*}][PF_6_] (*x* = 0, 1) [33,34,35]. The pattern of cell cycle arrest promoted by compound 1 is often accompanied by concomitant or subsequent apoptotic cell death induction. Indeed, Ann/PI double staining revealed an enhanced presence of cells in the early (Ann+/PI-) and late (Ann+/PI+) phases of apoptosis upon the applied treatment (Figure 5b). Apart from the dual role of cell death in tumor progression, apoptosis is still considered a vital process confronting cancer spreading and the main approach in the design of cancer treatment, thus qualifying Ir-based drugs as highly valuable for further investigation [60].

**Figure 6:**
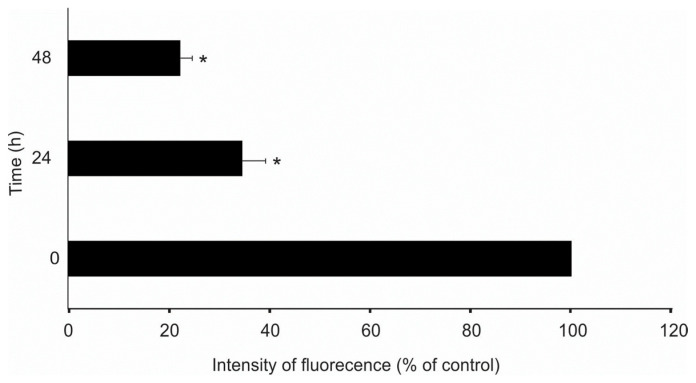
Effect of **1** on the ROS/RNS production in 8505C cells. Statistical significance * *p* < 0.05.

Caspases play a central role in driving apoptotic cell death by enzymatic cleavage of essential cellular proteins necessary for dismantling the dying cell [61]. However, caspase-independent apoptotic cell death is also observed. In addition, there is no linear correlation between the intensity of caspase activation and the apoptotic process. Complex **1** causes the moderate but significant activation of the caspase cascade detected by apostat assay (Figure 5c), resulting in robust apoptosis in 8505C cells. Before mentioned iridium(III) and ruthenium(II) complexes share the same pathway in tumoricidal action [33,34,35]. Furthermore, compound **1** does not affect significantly the presence of autophagic vesicles indicating that autophagy is not relevant as a destructive, nor protective process, in experimental drug action (Figure 5d). Importantly, the tested agent strongly downregulated the production of ROS/RNS in 8505C cells, measured by DHR (Figure 6). The importance of ROS in sustaining tumor viability highlights the potential efficacy of tumor suppression through the modulation of ROS levels by either reducing their production or enhancing their depletion [62]. For instance, metformin can activate the AMPK (5′-AMP-activated protein kinase) pathway, leading to an upregulation of thioredoxin, an antioxidant enzyme that effectively scavenges ROS, ultimately contributing to tumor eradication [63]. Accordingly, strong depletion of ROS/RNS upon treatment with **1**, compromised vitality of 8505C cells through elimination of reactive species involved in basic physiological processes responsible for cell growth.

## 4. Conclusions

Ph_2_PCH_2_CH_2_SPh was reacted with [Ir(η^5^-C_5_Me_5_)Cl_2_}_2_] dimer affording [Ir(η^5^C_5_Me_5_)Cl{Ph_2_P(CH_2_)_2_SPh-κ*P*,κ*S*}][PF_6_] (**1**). ^1^H, ^13^C and ^31^P NMR spectroscopy as well as SC-XRD confirmed the proposed coordination of the phospine-sulfide ligand. The main intermolecular stabilization interactions were H∙∙∙H contacts (49.7%) due to the abundance of hydrogen atoms in ligand and pentamethylcyclopentadienyl moiety. The correlation coefficient between experimental and theoretical bond lengths and angles was higher than 0.99, with a mean absolute error of 0.01 Å (bonds) and 1.0° (angles). The pseudo-octahedral geometry of the complex was restored upon optimization. Different donor–acceptor interactions were quantified between donor atoms and Ir(III) ion. The experimental ^1^H and ^13^C chemical shifts were well reproduced, with R and MAE values of 0.97 and 0.9 ppm (^1^H NMR)/0.99 and 4.1 ppm (^13^C NMR). The differences in these values were explained by the solvent effect. A viability assay was performed on MCF-7, SW480, 518A2, 8505C and A253 human tumor cell lines. Superior activity, compared to cisplatin, against MCF-7, SW480 and 8505C cell lines was observed for **1**. Intensive apoptosis together with cell cycle arrest in 8505C cells treated with **1** was observed. In parallel, a strong decrease in ROS/RNS was in correlation with the detected cytotoxic effect triggered by the experimental therapeutic. Further biological studies are advised based on these promising results.

## Figures and Tables

**Table 1:** Experimental and theoretical (at B3LYP-D3BJ/6-311++G(d,p)((H,C,P,S,Cl)/LanL2DZ(Ir) level of theory) ^1^H and ^13^C chemical shifts (in ppm).

^1^H NMR	Experimental	Theoretical	^13^C NMR	Experimental	Theoretical
C*H*_3_	1.50	1.69	C*C*H_3_	8.1	10.0
C*H*_2_PPh_2_	2.87	2.58	*C*H_2_PPh_2_	25.2	36.2
C*H*_2_PPh_2_	3.13	3.14	S*C*H_2_	34.7	40.8
SC*H*_2_	3.66	3.45	*C*CH_3_	96.9	96.3
SC*H*_2_	4.20	3.50	*C*Ph	96.9	96.3
HPh	7.12–7.70	7.06–8.18	R		0.99
R		0.97	MAE [ppm]		4.1
MAE [ppm]		0.9			

**Table 2:** IC_50_ values (mean ± SD, [µM]) of **1**, ligand Ph_2_P(CH_2_)_2_SPh, similar complexes and cisplatin obtained with SRB assays after 96 h treatment.

	MCF-7	SW480	518A2	8505C	A253	MRC-5
	IC_50_ [µM]					
Ph_2_P(CH_2_)_2_SPh [31]	6.3 ± 0.2	11.3 ± 1.2	20.5 ± 1.9	11.0 ± 1.2	9.5 ± 1.1	n.d.
[Ir(η^5^C_5_Me_5_)Cl{Ph_2_PCH_2_SPh-κ*P*,κ*S*}][PF_6_] [33]	16.0 ± 1.9	14.6 ± 3.4	9.8 ± 0.5	16.8 ± 3.5	10.2 ± 2.8	n.d.
[Ir(η^5^C_5_Me_5_)Cl{Ph_2_P(CH_2_)_2_SPh-κ*P*,κ*S*}][PF_6_]	1.3 ± 0.3	2.5 ± 0.5	1.3 ± 0.4	1.6 ± 0.2	1.7 ± 0.2	15.3 ± 2.3
[Ir(η^5^C_5_Me_5_)Cl{Ph_2_P(CH_2_)_3_SPhκ*P*,κ*S*}][PF_6_] [34,35]	0.4 ± 0.1	1.0 ± 0.3	1.0 ± 0.6	0.5 ± 0.2	0.4 ± 0.0	6.25 ± 0.31
[Ru(p-cym)Cl{Ph_2_P(CH_2_)_2_SPh-κ*P*,κ*S*}][PF_6_] [32]	0.26 ± 0.03	1.73 ± 0.14	1.70 ± 0.06	1.70 ± 0.06	0.93 ± 0.04	n.d.
**Cisplatin**	2.3 ± 0.2	3.0 ± 0.2	1.7 ± 0.2	5.5 ± 0.3	1.1 ± 0.2	0.65 ± 0.04

## Data Availability

Data are contained within the article and Supplementary Materials.

## Supplementary Materials

The following supporting information can be downloaded at: https://www.mdpi.com/article/10.3390/biom14040420/s1, Figure S1: Fingerprint plots of the most important contacts in the Hirshfeld surface analysis; Table S1: Crystal data and structure refinement for **1**; Table S2: Crystallographic and optimized (at B3LYP-D3BJ/6-311++G(d,p)( (H,C,P,S,Cl)/LanL2DZ(Ir) level of theory) bond lengths (numbers follow figure below); Table S3: Crystallographic and optimized at (B3LYP-D3BJ/6-311++G(d,p)( (H,C,P,S,Cl)/LanL2DZ(Ir) level of theory) bond angles; Table S4: Second order perturbation theory energies of stabilization interactions (B3LYP-D3BJ/6-311++G(d,p)( (H,C,P,S,Cl)/LanL2DZ(Ir) level of theory) within structure; Figure S2: Viability of primary human fibroblasts MRC-5 in response to the **1** (SRB assay, treatment 96 h).
