# Supplementary material for: (Pentamethylcyclopentadienyl)chloridoiridium(III) Complex Bearing Bidentate Ph2PCH2CH2SPh-κP,κS Ligand
Source: Biomolecules. 2024 Mar 30;14(4):420. doi: 10.3390/biom14040420 (PMC11048224; doi:10.3390/biom14040420)
Supplement: Supplementary file 1 [file biomolecules-14-00420-s001.zip › biomolecules-2886310-supplementary.pdf]

Supplementary information for:

(Pentamethylcyclopentadienyl)chloridoiridium(III) Complex Bearing bidentate $Ph_2PCH_2CH_2SPh$-*κP,κS* Ligand

Gerd Ludwig [1], Ivan Ranđelović [2, 3], Dušan Dimić[4], Teodora Komazec [2], Danijela Maksimović-Ivanić [2], Sanja Mijatović [2], Tobias Rüffer [5] and Goran N. Kaluđerović [6,*]

[1]Institute of Chemistry, Martin Luther University Halle-Wittenberg, Kurt-Mothes-Straße 2, D-06120 Halle, Germany;

[2]Department of Immunology, Institute for Biological Research "Sinisa Stankovic", University of Belgrade, Bulevar despota Stefana 142, 11060 Belgrade, Serbia;

[3] present affiliation: Department of Experimental Pharmacology, The National Tumor Biology Laboratory, National Institute of Oncology, Ráth György u. 7-9, 1122 Budapest, Hungary;

[4]Faculty of Physical Chemistry, University of Belgrade, Studentski trg 12-16, 11000 Belgrade, Serbia;

[5]Institute of Chemistry, Chemnitz University of Technology, Straße der Nationen 62, D-09111 Chemnitz, Germany;

[6]Department of Engineering and Natural Sciences, University of Applied Sciences Merseburg, Eberhard-Leibnitz-Strasse 2, 06217 Merseburg, Germany.

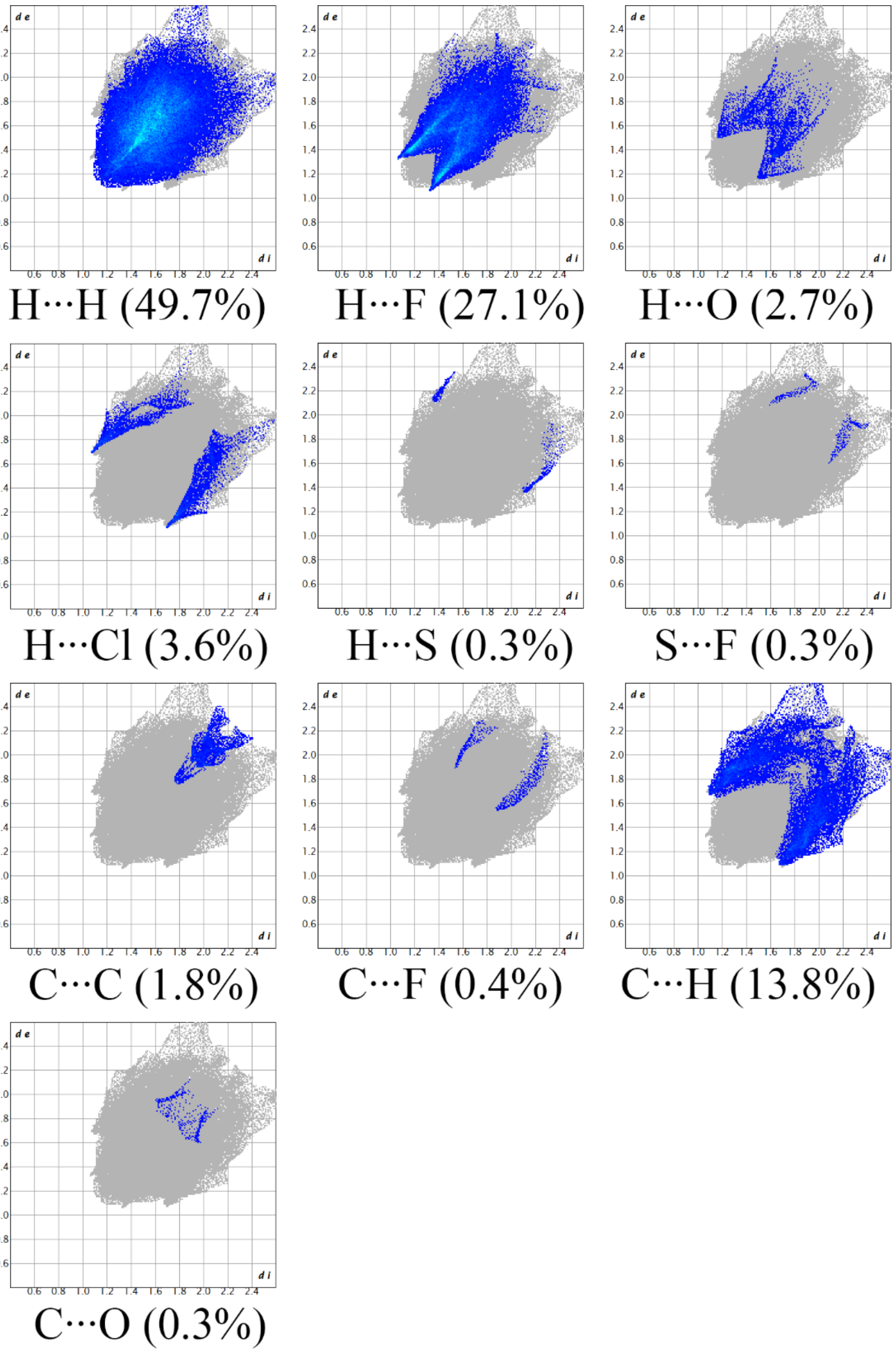

Figure S1. Fingerprint plots of the most important contacts in the Hirshfeld surface analysis.

Table S1. Crystal data and structure refinement for **1**

Empirical formula	$C_{33}H_{40}ClF_6IrOP_2S$
Formula weight	888.30
Temperature (K)	120
Wavelength (Å)	1.54184
Crystal system, space group	Triclinic
Space group	$P\bar{1}$
a (Å)	8.431(1)
b (Å)	12.387(2)
c (Å)	17.171(2)
α, β, γ (°)	100.306(9), 92.768(9), 107.18(1)
Volume (Å^3)	1675.7
Z	2
Calculated density (mg/mm^3)	1.761
Absorption coefficient (mm^{-1})	10.462
F(000)	880
Crystal size (mm)	0.3 × 0.2 × 0.2
θ range for data collection (°)	3.814 to 65.521
Limiting indices	$-9 \leq h \leq 9$, $-14 \leq k \leq 14$, $-20 \leq l \leq 12$
Reflections collected	9843
Reflections unique	5598 [R_{int} = 0.0279]
Data completeness (%)	92.3
Absorption correction	Semi-empirical from equivalents
Max. and min. transmission	1.00000 and 0.60742
Refinement method	Full-matrix least-squares on F^2
Data / restraints / parameters	5598 / 30 / 388
Goodness-of-fit on F^2	1.040
Final R indices [$I \geq 2\sigma(I)$]	R_1 = 0.0347, wR_2 = 0.0870
R indices (all data)	R_1 = 0.0372, wR_2 = 0.0883
Largest diff. peak and hole (e·A^{-3})	1.681 and -1.361

Table S2. Crystallographic and optimized (at B3LYP-D3BJ/6-311++G(d,p)((H,C,P,S,Cl)/LanL2DZ(Ir) level of theory) bond lengths (numbers follow figure below)

Bond	Experimental	Optimized
C1-C2	1.442(7)	1.44
C2-C3	1.437(7)	1.46
C3-C4	1.429(7)	1.41
C4-C5	1.458(7)	1.46
C5-C1	1.441(7)	1.44
C1-Ir1	2.211(4)	2.23
C2-Ir1	2.193(4)	2.21
C3-Ir1	2.234(4)	2.28
C4-Ir1	2.227(4)	2.28
C5-Ir1	2.216(4)	2.21

C2-C7	1.503(6)	1.49
C3-C8	1.489(6)	1.49
C4-C9	1.501(7)	1.49
C5-C10	1.488(7)	1.49
C1-C6	1.492(7)	1.49
Ir1-Cl1	2.393(1)	2.45
Ir1-P1	2.293(1)	2.32
Ir1-S1	2.350(1)	2.42
S1-C11	1.794(5)	1.80
C11-C12	1.387(7)	1.39
C12-C13	1.392(7)	1.39
C13-C14	1.392(8)	1.39
C14-C15	1.366(8)	1.39
C15-C16	1.393(8)	1.39
C16-C11	1.384(7)	1.40
S1-C17	1.847(5)	1.85
C17-C18	1.512(7)	1.52
C18-P1	1.834(4)	1.85
P1-C19	1.811(5)	1.82
C19-C20	1.399(7)	1.40
C20-C21	1.392(7)	1.39
C21-C22	1.384(7)	1.40
C22-C23	1.388(7)	1.39
C23-C24	1.378(7)	1.39
C24-C19	1.394(7)	1.40
P1-C25	1.827(5)	1.83
C25-C26	1.394(7)	1.40
C26-C27	1.394(7)	1.39
C27-C28	1.387(8)	1.40
C28-C29	1.362(8)	1.39
C29-C30	1.401(7)	1.39
C30-C25	1.391(7)	1.40
R	0.99	
MAE [Å]	0.01	

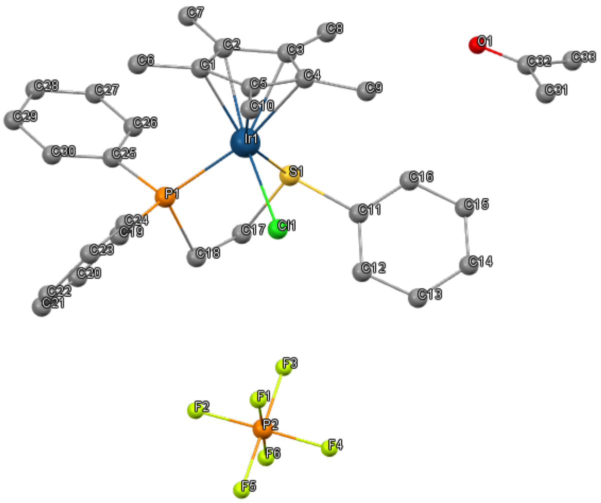

Table S3. Crystallographic and optimized at (B3LYP-D3BJ/6-311++G(d,p)((H,C,P,S,Cl)/LanL2DZ(Ir) level of theory) bond angles

Bond angle	Experimental	Optimized
C1-C2-C3	107.9(4)	107.5
C6-C1-C2	125.7(4)	126.0
C6-C1-C5	126.4(4)	125.8
C2-C3-C4	109.0(4)	108.3
C7-C2-C1	125.5(4)	126.5
C7-C2-C3	125.5(4)	124.6
C3-C4-C5	107.2(4)	108.3
C8-C3-C2	123.8(4)	124.9
C8-C3-C4	127.2(4)	126.7
C4-C5-C1	108.1(4)	107.6
C9-C4-C3	127.9(4)	127.1
C9-C4-C5	124.8(4)	124.6
C5-C1-C2	107.7(4)	108.0
C10-C5-C4	124.2(4)	124.3
C10-C5-C1	127.6(4)	126.8
C1-Ir1-C2	38.2(2)	37.9
C2-Ir1-C3	37.9(2)	37.8
C3-Ir1-C4	37.4(2)	36.1
C4-Ir1-C5	38.3(2)	37.9
C5-Ir1-C1	38.0(2)	37.9
C1-Ir1-Cl1	121.5(1)	134.5
C1-Ir1-P1	101.3(1)	99.7
C1-Ir1-S1	146.2(1)	133.3
Cl1-Ir1-P1	87.42(4)	86.8
Cl1-Ir1-S1	91.77(4)	91.9

P1-Ir1-S1	84.45(4)	85.4
S1-C11-C12	123.3(4)	123.4
S1-C11-C16	115.1(4)	115.8
C11-C12-C13	118.8(5)	119.2
C12-C13-C14	120.0(5)	120.4
C13-C14-C15	120.3(5)	120.0
C14-C15-C16	120.8(5)	120.1
C15-C16-C11	118.6(5)	119.5
C16-C11-C12	121.5(5)	120.8
C11-S1-C17	102.9(2)	103.4
S1-C17-C18	113.0(3)	113.6
C17-C18-P1	111.2(3)	112.3
C18-P1-C19	103.6(2)	103.8
C18-P1-C25	106.6(2)	106.4
P1-C19-C20	119.5(4)	120.2
C19-C20-C21	120.1(4)	120.0
C20-C21-C22	119.8(4)	120.1
C21-C22-C23	120.1(4)	120.0
C22-C23-C24	120.4(4)	120.1
C23-C24-C19	120.3(4)	120.2
C24-C19-C20	119.2(4)	119.7
C24-C19-P1	121.2(3)	120.1
C19-P1-C25	104.7(2)	105.4
P1-C25-C26	119.4(4)	119.3
C25-C26-C27	121.0(5)	120.5
C26-C27-C28	119.3(5)	120.1
C27-C28-C29	120.7(5)	119.8
C28-C29-C30	120.1(5)	120.3
C29-C30-C25	120.5(5)	120.2
C30-C25-C26	118.4(4)	119.2
C30-C25-P1	122.2(4)	121.5
R	0.99	
MAE [º]	1.0	

Table S4ss. Second order perturbation theory energies of stabilization interactions (B3LYP-D3BJ/6-311++G(d,p)((H,C,P,S,Cl)/LanL2DZ(Ir) level of theory) within structure.

Stabilization interaction	Energy [kJ mol^{-1}]
π(C-C)→ σ*(Ir-C)	67.5 – 370.0
π(C-C)→LP*(Ir)	19.9 – 88.9
π(C-C)→ π*(C-C)	8.9
$\pi(C\text{-}C)_{arom} \rightarrow \pi^*(C\text{-}C)_{arom}$	82.3 – 98.0
$\pi(C\text{-}C)_{arom} \rightarrow \sigma^*(Ir\text{-}P)$	14.5
$\pi(C\text{-}C)_{arom} \rightarrow \sigma^*(Ir\text{-}S)$	6.2

σ(Ir-C)→π*(C-C)	128.9
σ(Ir-C)→σ*(Ir-C)	125.1
σ(Ir-C)→σ*(Ir-P)	44.8
σ(Ir-C)→σ*(Ir-S)	123.8
LP(S) →LP*(Ir)	3.9
LP(S)→σ*(Ir-C)	4.9
LP(S)→π*(C-C)	22.7
LP(Cl)→LP*(Ir)	7.1
LP(Cl)→σ*(Ir-C)	17.6
LP(Cl)→σ*(Ir-S)	16.1
LP(Ir)→π*(C-C)	48.1
LP(Ir)→σ*(C-S)	6.0
LP(Ir)→σ*(C-P)	5.8

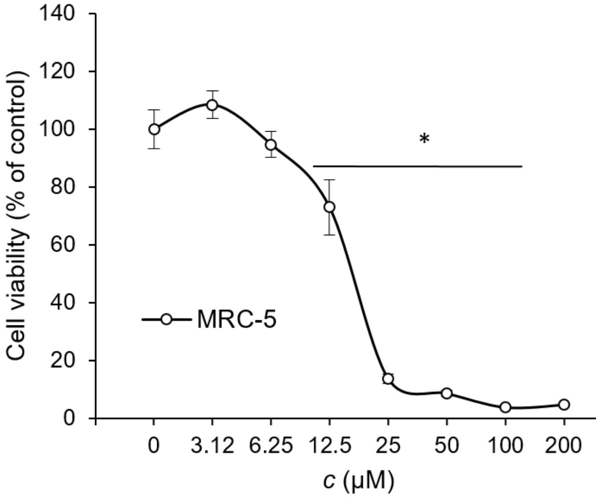

Figure S2. Viability of primary human fibroblasts MRC-5 in response to the **1** (SRB assay, treatment 96 h). Selectivity indices:

	IC$_{50}$ (MRC-5) / IC$_{50}$ (tumor cells)				
	**MCF-7**	**SW480**	**518A2**	**8505C**	**A253**
**1**	≈ 12	≈8	≈ 12	≈ 10	≈ 9
